# Probing key elements of teixobactin–lipid II interactions in membranes†
†Electronic supplementary information (ESI) available: Computational methods and supplementary figures. See DOI: 10.1039/c8sc02616e


**DOI:** 10.1039/c8sc02616e

**Published:** 2018-07-20

**Authors:** Po-Chao Wen, Juan M. Vanegas, Susan B. Rempe, Emad Tajkhorshid

**Affiliations:** a Department of Biochemistry , Center for Biophysics and Quantitative Biology , Beckman Institute for Advanced Science and Technology , University of Illinois at Urbana-Champaign , Urbana , Illinois 61801 , USA . Email: pwen2@illinois.edu ; Email: emad@life.illinois.edu; b Department of Nanobiology , Center for Biological and Engineering Sciences , Sandia National Laboratories , Albuquerque , NM 87185 , USA . Email: slrempe@sandia.gov

## Abstract

Teixobactin (Txb) is a recently discovered antibiotic against Gram-positive bacteria that induces no detectable resistance. The bactericidal mechanism is believed to be the inhibition of cell wall biosynthesis by Txb binding to lipid II and lipid III. Txb binding specificity likely arises from targeting of the shared lipid component, the pyrophosphate moiety. Despite synthesis and functional assessment of numerous chemical analogs of Txb, and consequent identification of the Txb pharmacophore, the detailed structural information of Txb–substrate binding is still lacking. Here, we use molecular modeling and microsecond-scale molecular dynamics simulations to capture the formation of Txb–lipid II complexes at a membrane surface. Two dominant binding conformations were observed, both showing characteristic lipid II phosphate binding by the Txb backbone amides near the C-terminal cyclodepsipeptide (d-Thr8–Ile11) ring. Additionally, binding by Txb also involved the side chain hydroxyl group of Ser7, as well as a secondary phosphate binding provided by the side chain of l-*allo*-enduracididine. Interestingly, those conformations differ by swapping two groups of hydrogen bond donors that coordinate the two phosphate moieties of lipid II, resulting in opposite orientations of lipid II binding. In addition, residues d-*allo*-Ile5 and Ile6 serve as the membrane anchors in both Txb conformations, regardless of the detailed phosphate binding interactions near the cyclodepsipeptide ring. The role of hydrophobic residues in Txb activity is primarily for its membrane insertion, and subsidiarily to provide non-polar interactions with the lipid II tail. Based on the Txb–lipid II interactions captured in their complexes, as well as their partitioning depths into the membrane, we propose that the bactericidal mechanism of Txb is to arrest cell wall synthesis by selectively inhibiting the transglycosylation of peptidoglycan, while possibly leaving the transpeptidation step unaffected. The observed “pyrophosphate caging” mechanism of lipid II inhibition appears to be similar to some lantibiotics, but different from that of vancomycin or bacitracin.

## Abstract 2

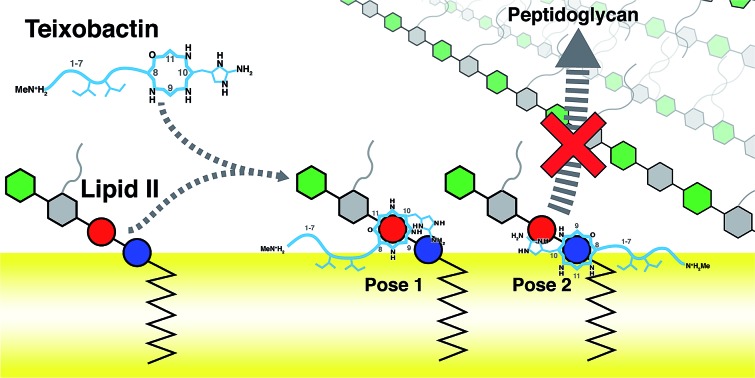
Two binding poses of the teixobactin–lipid II complex were captured with MD simulations at the membrane surface.

## Introduction

**Fig. 1:**
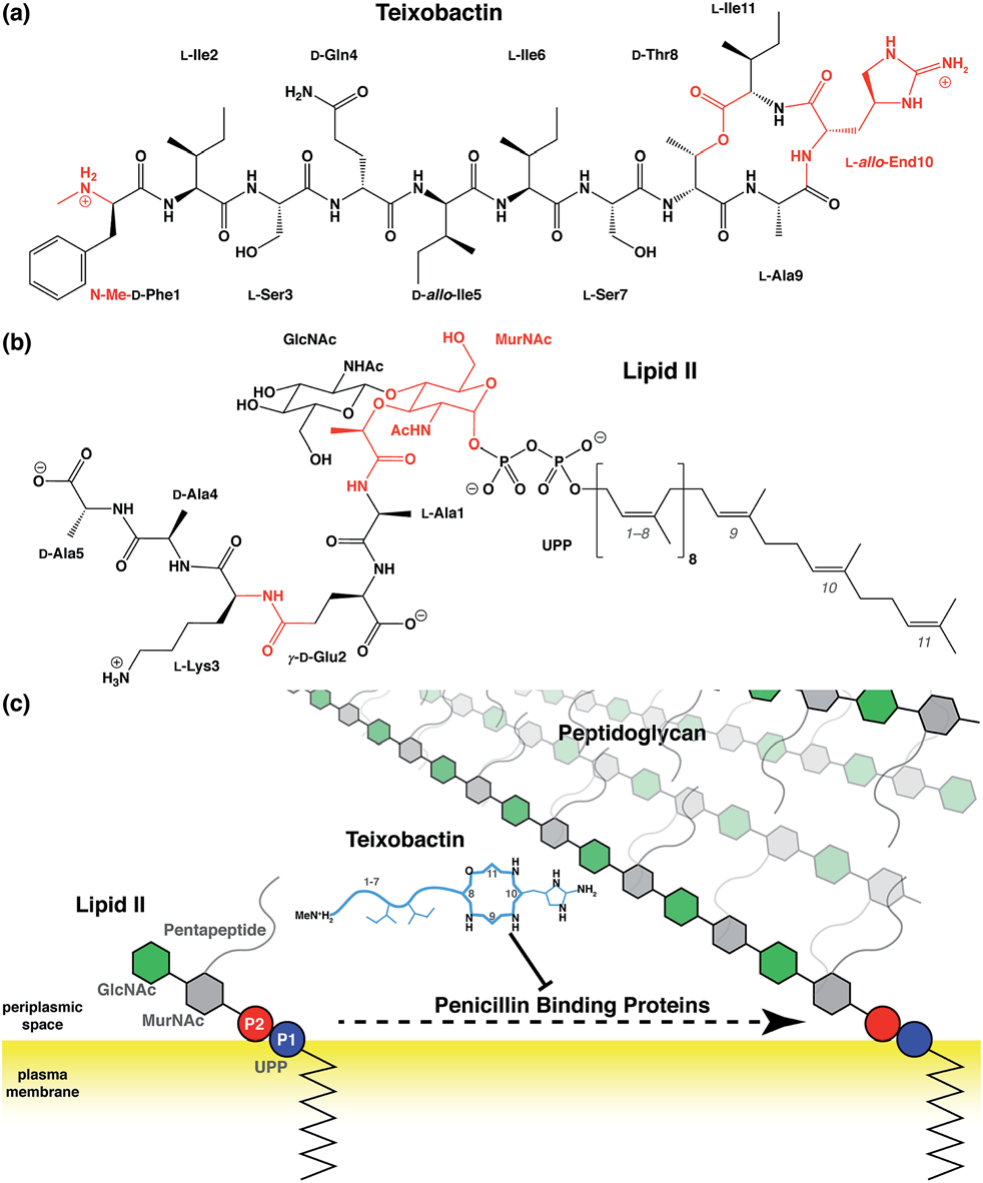
Chemical structures of (a) teixobactin and (b) lipid II; and (c) a schematic representation of the bactericidal mechanism. Chemical groups in (a) and (b) that require force field parameter developments are highlighted in red, and the isoprene subunits of lipid II are numbered from the phosphate end. (c) Components of lipid II are polymerized into peptidoglycan by penicillin binding proteins, a process that has been hypothesized to be inhibited by Txb through its direct interaction with lipid II. Chemical components in schematic presentations are shown as circles (phosphates), hexagons (pyranoses), and lines/curves (peptide chains). Relative residue numbering is labeled in Txb, and important side chains are drawn schematically. The surface of the bacterial plasma membrane is shown in yellow.

Antibiotic resistance has become a major threat to public health in recent years.^1^–^3^ Among many novel developments in antimicrobial agents,^4^,^5^ one of the recently discovered antibiotics isolated from *Eleftheria terrae*, named teixobactin (Txb, Fig. 1a), has shown promising bactericidal activities toward Gram-positive bacteria without inducing observable resistance.^6^ The bacteriolytic mechanism of Txb was shown to be the inhibition of multiple cell wall biosynthesis pathways,^7^ most importantly, by binding to the peptidoglycan precursor lipid II (Fig. 1b and c). Since Txb can inhibit directly multiple cell wall precursors that are all chemical derivatives of undecaprenylpyrophosphate (UPP), the binding interactions have been hypothesized to target specifically the common chemical moiety shared among its substrates: the pyrophosphate groups (P1 and P2).^6^

Despite characterization of its chemical structure, limited knowledge is available regarding the interactions between Txb and its target molecules. The Txb molecule is a depsipeptide consisting of 11 residues (Fig. 1a), of which 5 amino acids are non-proteinogenic. The latter comprise four d-amino acids and one l-*allo*-enduracididine (*allo*-End) at position 10. In addition, Txb contains an N-terminal methylation and a C-terminal “head-to-side-chain” cyclodepsipeptide ring.^6^ Most interactions have been interpreted based on the comparison of bacterial growth inhibition activities of numerous chemically synthesized Txb analogs.^8^–^27^ In particular, one of the inactive analogs, designated “Ac–Δ_1–5_–Arg_10_–Txb”,^15^ has been crystallized from solution as a hydrochloride salt, where the chloride anion is coordinated at the center of several backbone amides that form the C-terminal cyclodepsipeptide ring. The guanidinium group of the arginine side chain (which replaces the *allo*-End in wildtype Txb) also contributes to chloride binding.

As surmised from the chloride-bound crystal structure of this inactive Arg10-based analog, as well as the relative activities of other Txb analogs with single-residue substitutions, the binding of Txb to lipid II or other UPP derivatives is hypothesized to consist of two kinds of interactions: polar interactions that are primarily mediated by the cyclodepsipeptide ring, and hydrophobic interactions that are likely contributed by residues at the two termini.^15^ As the structure of the *bona fide* Txb–lipid II complex is yet to be determined, these hypothesized interactions remain to be elucidated. Additionally, since the crystallized Txb analog strongly favors binding to a chloride anion rather than to an inorganic pyrophosphate,^15^ it is possible that some key interactions of Txb are not reproduced by the inactive analog, or that certain interactions can take place only at a membrane surface and not in aqueous solution.

In agreement with the anion binding shown in the analog structure, a recent study proposed several binding modes between Txb and lipid II based on interactions captured with molecular dynamics (MD) simulations in aqueous solution.^28^ The majority of the interactions involved the binding of an anionic species (*e.g.*, a carboxylate or phosphate group of lipid II) by the cyclodepsipeptide ring. Despite a detailed view of Txb–lipid II interactions in solution, the previous modeling study does not consider the ionization state of Txb in aqueous solution, and the resulting binding modes do not explain the substrate selectivity of Txb, nor can they interpret or predict the activity of Txb analogs.

Here, we report the structural model of the Txb–lipid II complex obtained from extensive MD simulations in the presence of an explicit membrane. The Txb molecule is found to bind lipid II at the pyrophosphate in two orientations, each specifically coordinating one of the phosphates with the cyclodepsipeptide ring, and the other phosphate with the *allo*-End side chain. Although hydrophobic residues in Txb are essential for its activity, this requirement appears important generally for anchoring Txb at the membrane surface, rather than promoting a specific lipid II-binding mode involving hydrophobic interactions. The specific Txb–lipid II binding, and Txb-membrane binding interactions observed in this study are largely consistent with known structure–activity relationships of chemically synthesized Txb analogs, and may help in future design of Txb analogs that are easier to synthesize while retaining the excellent bactericidal activities of the wildtype molecule.

## Results

### Overview of the modeling strategy

**Fig. 2:**
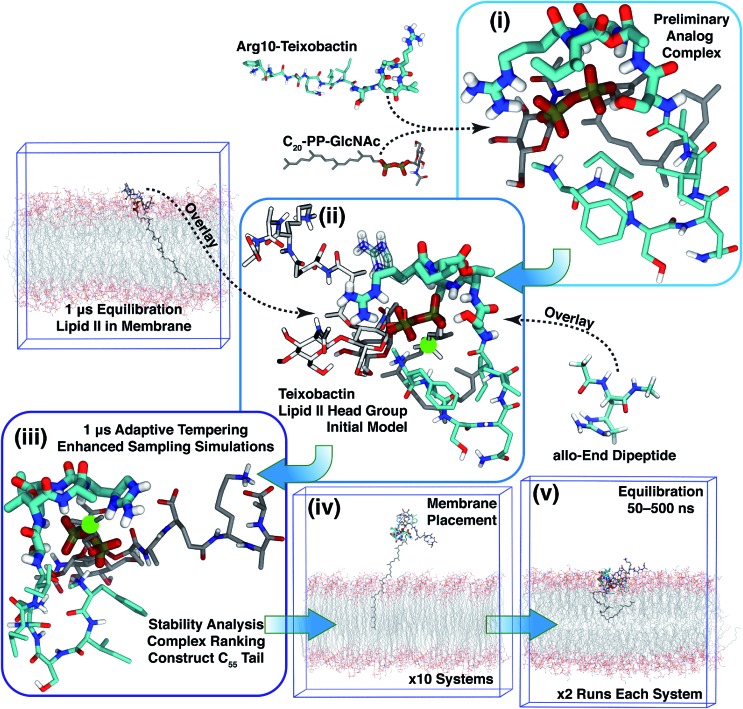
Diagram demonstrating the modeling strategy of this study. The general coloring scheme of atomic structures is: polar hydrogen (white), nitrogen (blue), oxygen (red), phosphorus (orange), carbon (cyan) (Txb) or gray (lipid II). Non-polar hydrogen atoms are not shown for clarity. Residues Ser7–Ile11 of Txb, and pyrophosphate of lipid II are highlighted in thicker sticks. For step ii, the structures adopted from step i are shown in the general coloring scheme, while the overlaid lipid II head group is shown in lighter colors, and the overlaid *allo*-End residue is shown in transparent sticks. The immobile atom in step iii is highlighted in a green sphere. Only membrane simulation systems are outlined at their periodic boundary boxes.

Based on Txb's substrate selectivity^6^ and proposed pharmacophore,^15^ Txb may bind lipid II at its head group. Then the most likely interactions would take place between the pyrophosphate of lipid II and the cyclodepsipeptide ring of Txb. To capture these possibilities, we carried out a stepwise modeling strategy (Fig. 2): (i) construction of a preliminary complex model based on two analogs (Arg10–Txb and 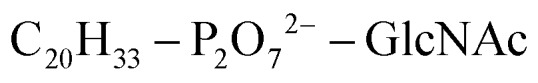) using MD simulations in aqueous solution; (ii) conversion of the analog complex in aqueous solution to an initial complex model of wildtype Txb and the head group of lipid II; (iii) exploration and screening of viable binding poses of the Txb–lipid II complex in aqueous solution using enhanced sampling simulations and accompanying analyses; (iv) addition of the full-length undecaprenyl (C_55_H_89_–) tail to the viable complexes obtained in step iii and insertion into an explicit membrane; (v) equilibration of the membrane-bound Txb–lipid II complex, and extended equilibrium simulations for the most stable complexes. The most promising Txb–lipid II binding poses obtained from membrane simulations are further analyzed and compared. The detailed procedure followed in each step is described in the ESI,† and the results of each step are described in the following sections.

### Preparative steps and initial modeling

To simulate Txb and lipid II using the CHARMM force field,^29^–^33^ topology and parameters are required for several chemical groups (Fig. 1a and b; highlighted in red). While parameters of all lipid II components and the ester group of Txb can be derived by referencing chemical analogs that are already available in CHARMM, parameters for the methylated N-terminus and the *allo*-End side chain of Txb were developed from *ab initio* calculations due to the lack of analogy in existing CHARMM parameter sets. Based on the p*K*_a_ of amino and guanidium groups, and the NMR assignment of Txb,^6^ both the methylated N-terminus and the *allo*-End side chain were modeled in their positively charged states, where the positive charges were anticipated to play important roles in binding to the negatively charged pyrophosphate group of lipid II.

To prepare for the simulation of Txb–lipid II complex formation at the membrane–water interface, a lipid II molecule was partially inserted into a 1-palmitoyl-2-oleoyl-phosphatidyl-ethanolamine (POPE) bilayer, chosen to represent bacterial membranes such as the inner membrane of *Escherichia coli*,^34^ or the plasma membrane of certain *Bacillus* species.^35^,^36^ The inserted lipid II molecule was simulated for 1 μs to capture its membrane-bound configuration. The initial structure of lipid II was modeled based on the solution NMR structure of the complex between the antibiotic nisin and a lipid II analog.^37^ First, the head-group coordinates were adopted and then the lipid tail was replaced with the canonical undecaprenyl group with its tip inserted into a POPE bilayer (similar to step iv in Fig. 2). The lipid II molecule was then equilibrated together with the newly constructed POPE bilayer. Insertion of the undecaprenyl tail led to rapid and full partitioning of the hydrophobic tail of lipid II so that the pyrophosphate group reached and fully partitioned into the membrane surface (the average phosphate plane of the *cis* leaflet, data not shown).

To create an initial model of the Txb–lipid II complex, the conformational ensemble of the membrane-bound lipid II collected in the μs-scaled simulation described above was superimposed onto a modeled complex of an Arg10–Txb mutant and a lipid III analog (which shares the core structure with lipid II), using the coordinates of the pyrophosphate group 
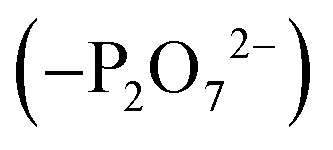
 for fitting according to the least root-mean-squared deviation (RMSD). Atomic contacts between Arg10–Txb and the superimposed lipid II head group (excluding the undecaprenyl tail beyond the first 5 carbons) were calculated to reject the superimposed lipid II conformations with significant steric clashes. With a cutoff at 1.8 Å, only one structure resulted that was bound to the pyrophosphate and compatible with the modeled Arg10–Txb. At the same time, the Arg10 side chain was replaced by the *allo*-End side chain using the conformation of an *allo*-End dipeptide minimized in electronic structure software and superimposed onto Arg10 using the backbone coordinates, thus creating an initial model of the complex between wildtype Txb and the head group of lipid II (with a shortened dimethylallyl tail; see Fig. 2, step ii).

### Sampling complex conformations in aqueous solution

**Fig. 3:**
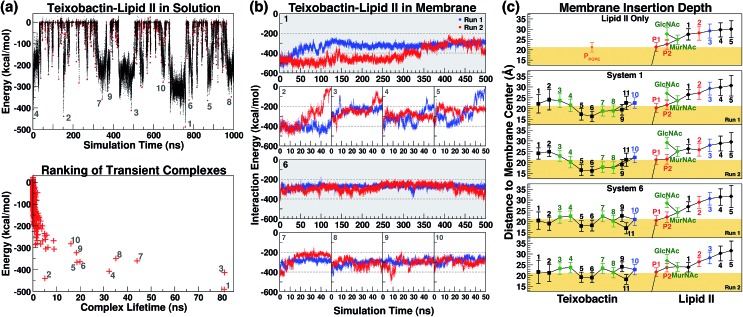
(a) The interaction energy between teixobactin and (tail-truncated) lipid II during the adaptive tempering simulations in aqueous solutions. The data were used to select the 10 highest-ranked complex conformations. The representative conformation of each teixobactin–lipid II encounter is marked as a red dot (top), and is ranked based on the interaction energy and the lifetime of the transient complex. (b) The teixobactin–lipid II interaction energy in the membrane simulations. (c) The positions of teixobactin/lipid II components relative to the membrane surface. Trajectories were re-centered to the center of the POPE bilayer and the adjusted *z*-coordinates were averaged over time. The membrane surface (yellow block) is defined by the averaged coordinates of POPE phosphorus atoms. Data points were labeled with the residue numbers of Txb and the lipid II pentapeptide, and the names of other components (P1/P2 for phosphates and MurNAc/GlcNAc for saccharides), with a color scheme applied to reflect the chemical properties of the components: hydrophobic (black), polar (green), and charged (blue for positive; red for negative). Lines were drawn to show covalent connectivity among components of Txb and lipid II. The line between P1 and the membrane center denotes the presence of the undecaprenyl tail.

The initial complex model described above was then subjected to 1 μs of enhanced sampling simulation in aqueous solution using the adaptive tempering method. During these simulations, the system temperature was varied between 298 K and 450 K according to the instantaneous total potential energy of the system.^38^ The iterative heating and cooling of the system resulted in repeated dissociation and re-binding of the two molecules. Counting the number of atomic contacts between Txb and lipid II using a 3 Å cutoff distance, a total of 1644 discrete encounters between the two molecules were observed within the 1 μs enhanced sampling trajectories (Fig. 3a). Each encounter was defined as a period with non-zero Txb–lipid II contacts separated from other encounters by periods of zero contact. A representative conformation of the Txb–lipid II binary complex was then selected for each of the 1644 encounters based on the time point of the strongest interaction (lowest potential) energy (Fig. 3a).

Even though numerous encounters occurred during the enhanced sampling simulations, the majority of them may fail to generate a stable complex configuration. Therefore, the most stable (and likely most relevant) encounters were identified by comparison of interaction energy for representative conformations, and the life time of the transient complexes generated by the encounter. Using these two criteria, only 10 encounters fulfilled the condition that either the complex (a) lasted for more than 10 ns under the adaptive tempering simulation conditions, or (b) reached a Txb–lipid II interaction energy more favorable than –400 kcal mol^–1^ (Fig. 3a, bottom). The interaction energy of these 10 selected conformations suggests that the nature of their interactions is highly polar or even ionic, consistent with the opposite net charges of the two molecules under pH = 7 (+2*e* for Txb and –3*e* for lipid II). Examining inter-molecular hydrogen bonds among the 10 binary complexes obtained from the enhanced sampling simulations, it became noticeable that lipid II binding of Txb is characterized by heavy involvement of the amide groups at the C-terminal cyclodepsipeptide ring (d-Thr8–Ala11) and the *allo*-End side chain, and to a lesser degree the charged, methylated N-terminus. On lipid II, most of those 10 complexes primarily involved interactions with the two phosphate groups, and occasionally the two carboxylate termini (γ-d-Glu2 and d-Ala5) of the pentapeptide (Fig. S1 and S3†).

### Formation of the teixobactin–lipid II complex in membranes

The 10 conformations of Txb–lipid II head group complexes in solution, described above, were ranked from 1 to 10 based on their interaction energies (Fig. 3a) and used to generate full lipid II complexes by extending the dimethylallyl group into an undecaprenyl tail, which was then inserted into a POPE bilayer (Fig. 2, step iv). Each of the 10 newly created systems was then equilibrated for 50 ns in two separate runs (replicas). At the end of the 50 ns runs, all of the lipid II tails had partitioned fully into the membrane, with the head groups reaching the membrane surface.

The same membrane partitioning did not always occur for the Txb molecules due to the dissociation of Txb from lipid II in some runs (*e.g.*, Run 2 of System 2 and Run 1 of System 5). Inspecting the interaction energy between Txb and lipid II in those 20 simulation runs, it was found that a significant portion of Txb–lipid II interactions in every system were lost upon full membrane association (Fig. 3b). For instance, comparing intermolecular hydrogen bonds between *t* = 0 and *t* = 50 ns in each of the 20 runs (Fig. S1†), it is clear that only the interactions involving phosphate binding—either from Txb backbone amides or side chains of Ser7/*allo*-End10—tend to be retained after 50 ns of simulations. All other polar interactions are displaced during the process of membrane partitioning, even for those forming salt bridges in the initial conformation (*e.g.*, between the N-terminus of Txb and one of the carboxylate groups of the pentapeptide). It is likely that all lipid II-binding interactions of Txb are subject to competition from POPE head groups during membrane partitioning, and thus only the most robust interactions (which mark the lipid II selectivity) survive equilibration in the explicit membrane environment.

Based on the Txb–lipid II interactions, complexes 1 and 6 were the two highest-ranked systems that retained most of the interactions at *t* = 50 ns (energy below –200 kcal mol^–1^ in both runs), while Systems 2–5 all had at least one run reaching higher energy conformations during the 50 ns (Fig. 3b). This observation suggests that Systems 1 and 6 might preserve the most essential Txb–lipid II interactions, and thus be worthy of further investigation. Even though the detailed atomic interactions between Txb and lipid II differ in Systems 1 and 6 (Fig. S1†), both depicted a common binding mode that lasted for 50 ns: phosphate binding by the backbone amides of the cyclodepsipeptide ring, as well as by the *allo*-End side chain. Interestingly, the lipid II-pyrophosphate in the two systems was bound in opposite orientations by these two groups of hydrogen bond donors. The cyclodepsipeptide ring binds only to the saccharide-connecting phosphate (P2) in System 1 while it binds only to the polyprenyl-connecting phosphate (P1) in System 6. The reverse is true for the *allo*-End side chain.

To investigate the long-term stability of these two types of lipid II binding interactions in membranes, all 4 simulation runs of Systems 1 and 6 were extended to 500 ns and their intermolecular interactions were analyzed further. At *t* = 500 ns, all the Txb molecules in the 4 simulation runs of Systems 1 and 6 stayed bound to lipid II, despite the variations in their intermolecular interactions over time. Examining the simulation trajectories, it was found that the two binding partners are highly dynamic in nature and form numerous transient contacts throughout their individual chemical components, although most contacts are too short-lived to be considered significant.

**Fig. 4:**
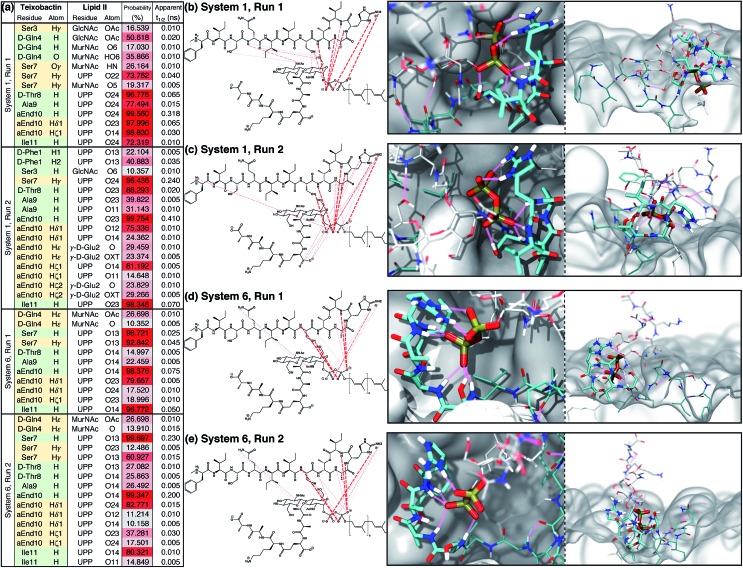
Teixobactin–lipid II binding in the membrane and the hydrogen bonds maintaining the complex. (a) List of hydrogen bonds between teixobactin and lipid II that have a significant presence (>10% occurring probability) in membrane simulations Systems 1 and 6, where the complex appeared stable for 500 ns. Hydrogen bonds contributed from the backbone and side chains of teixobactin are listed in green and yellow cells, respectively. The probability of hydrogen bonds occurring is also color-coded from gray (10%) to red (100%). For clarity, residue l-*allo*-End10 is shortened to aEnd10 here. See Fig. S2† for atom names in *allo*-End, UPP, MurNAc and GlcNAc. (b–e) Hydrogen bonds listed in (a) plotted over the chemical structures and colored based on the probability of occurring using the same color code as in (a); and *t* = 500 ns snapshot of the complex viewed from perpendicular (left) or parallel (right) to the membrane surface. The general coloring scheme of atomic structures is identical to the one described in Fig. 2, while hydrogen bonds are shown in pink lines and the membrane in white surfaces.

An extensive analysis of intermolecular hydrogen bonds between Txb and lipid II was conducted for all 4 trajectories between *t* = 50 ns and *t* = 500 ns. The analysis revealed that only ∼10% of the hydrogen bonds formed between the two molecules in each run exist for >10% of the simulation time. These hydrogen bonds are listed and plotted in Fig. 4. It is clear that the hydrogen bonds can be formed by completely different pairs of atoms, even when the two simulation trajectories are evolved from the very same initial structure (*e.g.*, between the two runs of System 1, Fig. 4b and c). Nevertheless, the phosphate-coordinating hydrogen bonds are always contributed mainly by the backbone amides of the cyclodepsipeptide ring, as well as the side chains of Ser7 and *allo*-End10. It is noteworthy that the cyclodepsipeptide ring amides and the residue Ser7 tend to bind to the same phosphate group, while the *allo*-End10 side chain tends to bind the other phosphate. This common trend suggests that the subtle differences in pairwise interactions demonstrated in runs of Systems 1 and 6 are, in fact, slight variations of the same binding mode. Similar analysis was also done to identify hydrophobic/non-polar contacts formed between Txb and lipid II in these 4 simulation runs. The infrequent non-polar Txb–lipid II contacts along with their inconsistent pattern among different simulation runs (Fig. S4†) suggest that the binding between Txb and lipid II is mainly maintained by polar interactions, which is consistent with the large interaction energy shown in Fig. 3b.

The Txb–lipid II binding interactions appear highly dynamic, yet robust. The median lifetime of the hydrogen bonds recorded in Systems 1 and 6 is all well below 1 ns, regardless of their probability of occurrence (Fig. 4a). This means that the hydrogen bonds between Txb and lipid II are constantly swapping with one another, likely due to the rotation of the phosphate groups and the peptide backbone. Or those hydrogen bonds get displaced transiently by water or lipid head groups. The complex conformation is nonetheless maintained by multiple simultaneous hydrogen bonds as at least 4 of them exist with >80% occurrence identified in each of the four runs (Fig. 4). Thus, a transient displacement of one hydrogen bond does not lead to dissociation of the complex. Instead, the short lifetimes of those hydrogen bonds further support the dynamic nature of the complex.

Membrane partitioning of the undecaprenyl tail of lipid II results in the localization of both Txb and the head group of lipid II near the membrane surface. The membrane insertion depths of Txb/lipid II components were averaged for the *t* = 50–500 ns time span of the 4 simulation runs of Systems 1 and 6 (Fig. 3c). As a control, the same analysis was also applied to the isolated lipid II simulation between *t* = 50 ns and *t* = 1 μs.

The results indicate that Txb is bound to the membrane surface upon complex formation; however, the cyclodepsipeptide ring can insert into the membrane with two opposite orientations (Fig. 4b–e). In System 1, residue Ala9 faces the membrane interior while Ile11 faces the solution. In System 6, in contrast, residue Ile11 appears to anchor to the membrane while Ala9 becomes exposed to the aqueous solution. It should be noted that the membrane binding orientation of the cyclodepsipeptide ring does not correlate with the phosphate binding orientation discussed above, as other combinations of membrane/phosphate binding orientations were observed among the other 16 simulation runs of 50 ns length. Instead, the membrane binding orientation of the cyclodepsipeptide ring is likely dictated by how the Txb molecule initially contacts and inserts into the membrane, while both orientations are capable of binding to the lipid II pyrophosphate.

More importantly, regardless of the membrane inserting orientation of the cyclodepsipeptide ring, residues d-*allo*-Ile5 and Ile6 are always partitioned deepest into the interior of the membrane (Fig. 3c). Although half of the residues in Txb are hydrophobic, only those two residues consistently showed membrane insertion in different lipid II binding poses. In contrast, the two N-terminal hydrophobic residues (d-Phe1 and Ile2) tended to partition closer to the membrane surface, while the two hydrophobic residues near the C-terminus (Ala9 and Ile11) were mutually exclusive in terms of their membrane insertion. This exclusivity occurred due to the locations of Ala9 and Ile11 at opposite corners of the four-residue cyclodepsipeptide ring. Thus, d-*allo*-Ile5 and Ile6 are the most likely candidates for the main membrane anchoring mechanism of Txb.

In contrast to the hydrophobic residues of Txb, the positions of lipid II components relative to the membrane surface appeared unaffected when bound to Txb. In general, the prenyl-connecting phosphate, P1, always lined up with the phosphates of other phospholipids at the membrane surface, with every additional chemical component away from P1 positioned slightly farther from the surface (Fig. 3c and 4b–e). This organization suggests that only the undecaprenyl tail and the pyrophosphate moiety of lipid II are truly membrane embedded, whereas the disaccharide and pentapeptide can be considered “add-on structures” of the membrane that are largely solution exposed. When the cyclodepsipeptide ring of Txb coordinates the saccharide-connecting phosphate, P2, (as in System 1, Fig. 4b and c), the binding results in a slight “sinking” of P2 toward the membrane surface (Fig. 3c). Nevertheless, the relative positions of all add-on structures appeared unaltered. This observation suggests that Txb binding to lipid II does not require a significant repartitioning of lipid II from its fully membrane-bound form. The highly dynamic nature of the lipid II add-on structures in solution, and their proximity to the membrane surface, do facilitate their frequent contact with the head groups of phospholipids, and occasionally with the pyrophosphate-bound Txb. Nevertheless, the interactions of Txb with lipid II disaccharide/pentapeptide groups are too transient to warrant further investigation.

## Discussions

### Lipid II-binding poses of teixobactin

**Fig. 5:**
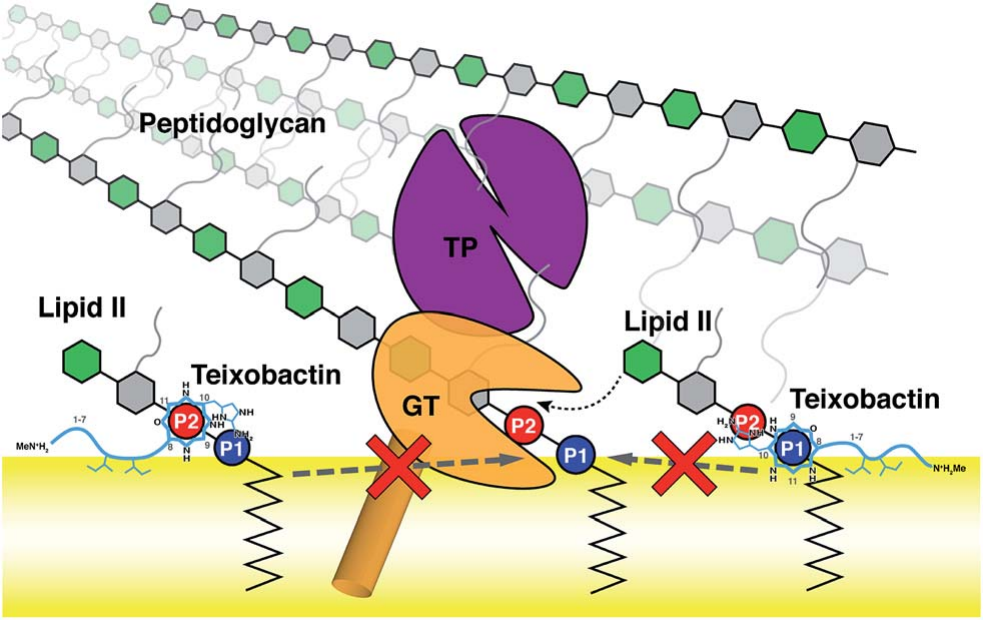
Schematic representation of the two poses of teixobactin–lipid II complexes at the membrane surface, and the proposed mechanism of peptidoglycan biosynthesis inhibition. A prototypical penicillin binding protein is shown as a combination of the membrane anchor (orange cylinder), the glycosyltransferase domain (GT, orange), and the transpeptidase domain (TP, purple).

Using the large dataset generated by MD simulations that started from diverse initial configurations, we obtained two lipid II binding poses of Txb that are stable in the membrane for at least 500 ns (Fig. 4, schematically shown in Fig. 5). Both poses led to a common set of functional annotations of Txb residues (Table 1): (i) residues Ser7–Ile11 coordinate the pyrophosphate group of lipid II, mainly using their backbone amides, while Ser7 and *allo*-End10 also participate in pyrophosphate binding through side chain hydrogen bonds; (ii) the residues d-*allo*-Ile5 and Ile6 act as the main membrane anchoring residues, where the membrane insertion is aided by (iii) one of the hydrophobic residues at the cyclodepsipeptide ring (Ala9 or Ile11), depending on the orientation in which Txb comes into contact with the membrane surface. Due to the close proximity of these hydrophobic residues to the phosphate-coordinators, some of these hydrophobic side chains also make frequent non-polar contacts with the tail of lipid II (Fig. S4†). The two poses differ mainly by the Txb phosphate-binding orientations at the cyclodepsipeptide ring. In one orientation, Txb coordinates the polyprenyl-connecting P1, and the saccharide-connecting P2 in the other orientation. Due to finite simulation time and limited conformational sampling, it is unclear whether the two conformations can interconvert without completely dissociating from lipid II.

**Table 1:** Summary of essential residues of Txb and their roles in lipid II binding

Residue	Group	Function
d-*allo*-Ile5	Side chain	Membrane anchoring
Ile6	Side chain	Membrane anchoring & prenyl binding
Ser7	Backbone	Phosphate binding
Ser7	Side chain	Phosphate binding
d-Thr8	Backbone	Phosphate binding
Ala9	Backbone	Phosphate binding
Ala9	Side chain	Membrane anchoring & prenyl binding^*a*^
*allo*-End10	Backbone	Phosphate binding
*allo*-End10	Side chain	Secondary phosphate binding
Ile11	Backbone	Phosphate binding
Ile11	Side chain	Membrane anchoring & prenyl binding^*a*^

^*a*^Membrane anchoring and prenyl binding by Ala9 or Ile11 are mutually exclusive.

Additional poses for the Txb–lipid II complex may exist since μs-scaled all-atom MD simulations do not sample exhaustively the conformational space of such a complex system. Nevertheless, the two poses captured by our simulations are largely consistent with experimentally determined characteristics of Txb: (a) the specific binding interactions are highly localized at the pyrophosphate of lipid II, consistent with the broad inhibition of UPP derivatives by Txb;^6^ (b) phosphate binding of Txb is provided exclusively by backbone amides near the C-terminal cyclodepsipeptide ring and the side chains of Ser7 and *allo*-End10, consistent with the proposed Txb pharmacophore from studies of several partially active Txb analogs;^9^,^15^ (c) residues d-*allo*-Ile5 and Ile6 serve as membrane anchors and all residues further up toward the N-terminus have little interaction with lipid II, consistent with the finding that the first 5 residues of Txb could be truncated while preserving partial activity;^9^,^15^ (d) lipid II binding does not deplete all the hydrogen bond donors of the *allo*-End10 side chain, implying that the guanidinium group is nonessential.

The latter observation is consistent with many *allo*-End10 substituted Txb analogs that retain partial or full antimicrobial activities compared with the wildtype Txb.^8^–^27^ Nevertheless, residue 10 of Txb remains an essential residue, regardless of the side chain identity, because of its critical role in phosphate binding by the backbone amide (∼99% probability of donating a hydrogen bond to lipid II, Fig. 4a). Evidence for the critical role of residue 10 backbone comes from amino acid substitutions that alter backbone conformations. For example, proline^23^ and d-amino acids^22^,^23^ at residue 10 result in significant reduction of Txb activity, sometimes even more than side chain substitutions that contain no hydrogen bond donor.

In our simulations, the hydrogen bond acceptors of the *allo*-End10 side chain usually belong to a different phosphate group from the one coordinated by the backbone amides (Fig. 4), indicating that the main phosphate-coordinators of Txb are the backbone amides around the cyclodepsipeptide ring (Ser7–Ile11) and perhaps also the side chain of Ser7. In contrast, the side chain of *allo*-End10 only serves as a secondary phosphate-coordinator that recognizes another phosphate group not bound to the backbone amides.

Interestingly, recent discoveries of several highly active Txb analogs^22^–^24^ all contain *allo*-End10 substitutions with medium-sized hydrophobic residues, suggesting that this secondary phosphate coordinating group at the residue 10 position is not absolutely required. Introduction of another d-Arg4 substitution of d-Gln4 in the context of Leu10 substitution of *allo*-End10, however, further reduces the minimal inhibitory concentrations of the Txb analog.^24^ Therefore, we speculate that the role of this d-Arg4 residue is also as a positively charged secondary phosphate-coordinator, presumably one that binds the phosphate group farther away from the cyclodepsipeptide ring.

In contrast to the side chain of *allo*-End10, which preferably binds a secondary phosphate group, the side chain of Ser7 mostly binds the same phosphate group that is coordinated by the cyclodepsipeptide backbone amides (Fig. 4a). This observation coincides with the loss of Txb-analog activity whenever Ser7 is substituted,^11^,^15^,^17^,^20^,^22^ suggesting that phosphate coordination by the side chain of Ser7 is also essential.

### Membrane insertion of teixobactin

Lipid II and other bactoprenol derivatives account for only ∼1% of total bacterial cellular lipids,^39^ and therefore it is likely that Txb would anchor to the bacterial plasma membrane and interact with the phospholipids before reaching a lipid II molecule. This means hydrophobic interactions are expected to play significant roles in Txb's activity. Granted, most known Txb analogs with substituted mid-chain hydrophobic residues lose their antimicrobial activities,^11^,^15^,^16^,^22^ indicating that hydrophobic interactions are indeed essential for Txb–lipid II binding.

In our simulations, although some hydrophobic residues of Txb do show direct hydrophobic contacts with lipid II (Fig. S4†), these hydrophobic interactions appear far less frequent than their polar counterparts. Therefore, the significance of membrane insertion by these hydrophobic residues clearly outweighs their direct lipid II binding. For example, several backbone amides of Txb show hydrogen bonding to lipid II phosphates at >90% probability (Fig. 4a), whereas the most prevailing hydrophobic contacts between Txb and lipid II rarely exceed 90% occurrence (Fig. S4†). Moreover, those hydrophobic residues with frequent lipid II contacts are either in close proximity to a phosphate coordinator (Ile6) or being one themselves (Ala9/Ile11), implying their hydrophobic interactions with lipid II are merely a secondary effect of the phosphate coordination. On the other hand, the consistently deep membrane insertion exhibited by these hydrophobic residues (Fig. 3c) manifests their primary role as membrane anchors rather than specific hydrophobic binding to lipid II.

Two distinct membrane-binding modes were captured in Systems 1 and 6, highlighted by the difference of membrane insertion depths at the N- and C-terminal residues. The two membrane binding modes also coincide with a slight, but noticeable, change in the relative positions of the two lipid II phosphates at the membrane surface (Fig. 3c). Lipid II phosphates in System 6 appear to be more in line with their relaxed (equilibrium) positions in an isolated lipid II, whereas a slight shift of the disaccharide-connecting P2 toward the membrane center can be observed in System 1. The latter is likely due to the P2-coordination by the cyclodepsipeptide ring.

Additionally, the membrane binding mode in System 6 also allows all hydrophobic side chains of Txb to partition in the interior of the phospholipid bilayer (except Ala9), a pattern that can be expected for an isolated, membrane bound Txb molecule. In contrast, membrane insertion depths in System 1 showed some hydrophobic mismatch at residues 1–2 and 7–8. Positioning of Txb and lipid II components all suggests that the membrane insertion depicted in System 6 might be more relevant to antimicrobial activity. This conclusion is further supported by studies of Txb analogs in which polar substitutions can be tolerated for Ala9 (ref. 15 and 17 and 27) but not Ile11.^15^,^27^ In contrast, some hydrophobic substitutions of Ile11 retain partial activity.^15^,^16^,^27^ Both results indicate that Ile11 is more likely the membrane anchor of the cyclodepsipeptide ring than Ala9 *in vivo*, matching the membrane binding mode described in System 6.

In addition to d-*allo*-Ile5, Ile6 and Ala9/Ile11, residues N–Me–d-Phe1 and Ile2 also come into contact with the membrane surface (Fig. 3c), especially in System 6 where Txb inserts in a presumably ideal orientation. Although we do not observe significant lipid II interaction outside the cyclodepsipeptide ring, it is possible that the membrane binding of these N-terminal residues is also required for the activity of Txb. This conclusion is supported by Txb analogs with a neutralized N-terminus or reduced side chain hydrophobicity at residue 1, all of which show reduced activities.^10^,^12^,^16^–^18^,^20^ Increasing the side chain hydrophobicity of residue 1, on the other hand, has been shown to boost the activity of this Txb analog.^26^

The role of Txb N-terminal residues in membrane binding may be underestimated in our simulations due to the membrane environment used. The bacterial membrane in this study is modeled as a simple bilayer of pure POPE; however, this zwitterionic phospholipid is not a major component in cell membranes of many Gram-positive bacteria.^36^,^40^ For example, the cell membrane of *Staphylococcus aureus*, the Gram-positive bacteria used often to test Txb analog activities, consists almost exclusively of anionic phospholipids such as phosphatidylglycerol and cardiolipin.^41^ When Txb binds to a bilayer of negatively charged lipids, a stronger lipid interaction from its positively charged N-terminus should be expected, which may result in a deeper insertion of the N-terminal residues regardless of the membrane binding orientation at the C-terminal cyclodepsipeptide ring. In other words, the stronger N-terminal interaction may obscure differences between membrane binding modes at the C-terminus.

We note that binding between Txb and lipid II is unlikely to depend on the high concentration of anionic lipids that occurs in model Gram-positive organisms. This assertion is supported by the observation that wildtype Txb retains moderate activity against *Escherichia coli* when its outer membrane is permeabilized,^6^ and its cellular lipids are predominantly phosphatidylethanolamines (PE).^34^

### Interpretations of the 2 : 1 ternary complex

The binding stoichiometry of Txb and its analogs to lipid II, or other UPP derivatives, has been reported as 2 : 1 at full capacity.^6^,^22^,^26^ A ternary complex of two Txb molecules and one lipid II was modeled recently, with Txb binding at both the pyrophosphate and the carboxylate of the pentapeptide of lipid II.^28^ We argue that the formation of a 2 : 1 Txb–lipid II ternary complex should be centered at the pyrophosphate moiety instead of the carboxylates of the pentapeptide. If half of the Txb was bound to the pentapeptide of lipid II, we should expect the same binding mode to take place at the peptidoglycan cell wall because free carboxylate groups also exist in uncross-linked peptidoglycan strands. In experiments, Txb does not interact with peptidoglycan,^7^ suggesting that the interaction between Txb and the lipid II pentapeptide is highly unlikely to contribute a major binding mode.

In contrast, the binding poses depicted in Systems 1 and 6 of our simulations are compatible with the formation of a pyrophosphate-centered ternary complex, in which each Txb molecule encages one of the two phosphates of lipid II from opposite sides of the pyrophosphate. A preliminary superpositioning of trajectories from Systems 1 and 6 leads to steric clashes between the two Txb molecules of either system, but most of the steric clashes take place at the *allo*-End10 side chain. Therefore, we hypothesize that the role of this secondary phosphate-coordinator is optional, and the side chain may disengage from the pyrophosphate to make room for another Txb cyclodepsipeptide ring upon the formation of a ternary complex.

The apparent 2 : 1 stoichiometry may also be an artifact from measuring Txb–lipid II binding in aqueous solution, where the solubility of either compound is low. The binding between a potent Txb analog and a soluble lipid II analog has been quantified in aqueous solution using isothermal titration calorimetry, where the apparent affinity constant *K*_d_ was measured at 23 μM with 2 : 1 stoichiometry.^26^ Similarly, the apparent *K*_d_ between another Txb analog and a different soluble lipid II analog was measured at 138 μM using quantitative NMR analysis.^22^ Both numbers are two to three orders of magnitude higher than the minimal inhibitory concentration of Txb (∼0.25 mg L^–1^,^6^ or ∼200 nM). Even though these analogs do show lower activities than wildtype Txb, those differences do not account for the more than 10-fold reduction of binding affinities.

The simple binding measurement in aqueous solution gets further complicated when the same Txb analog showing 23 μM apparent *K*_d_ was tested with lipid II instead of a more soluble analog. Then the full binding capacity extended beyond a 4 : 1 ratio.^26^ Together, these results suggest that measurements of binding in aqueous solutions are limited by the solubility of the compounds. Furthermore, the lack of a membrane environment may account for a loss of affinity and a bias in apparent stoichiometry.

The affinity and stoichiometry of Txb–lipid II binding can be compared with those of the lantibiotic nisin. The minimal inhibitory concentrations of nisin, when calculated in molar concentrations, fall within a sub-micromolar range.^42^,^43^ This range corresponds to the same order of magnitude as wildtype Txb. The binding of nisin to cell wall precursors was also measured at a 2 : 1 stoichiometry using aqueous/butanol extraction,^44^ the same method used to determine Txb–lipid II stoichiometry.^6^ The nisin–lipid II complex structure was solved with solution NMR spectroscopy.^37^ Despite the 1 : 1 binary complex found for nisin complexes, significant similarity in atomic details can be drawn with the Txb–lipid II complexes revealed in this study (described below).

Unlike Txb, however, multiple studies have measured the binding affinity of nisin with its substrates in membrane environments. In membranes, the apparent *K*_d_ all fall in the sub-micromolar range,^44^,^45^ agreeing with its minimal inhibitory concentrations. This agreement contrasts with the orders of magnitude in discrepancy between solution-based *K*_d_ and minimal inhibitory concentrations of Txb.^22^,^26^ Since Txb is known to work at a much lower concentration than the apparent *K*_d_ obtained in aqueous solution, it is possible that a 1 : 1 Txb–lipid II binary complex could be the functionally relevant form *in vivo* that inhibits peptidoglycan biosynthesis.

Note that the apparent *K*_d_ of the nisin–lipid II complex was derived from its *k*_on_ and *k*_off_ using the 1 : 1 binding formulation,^44^ implying that the formation of a ternary complex may not occur in the membrane of a limited area, and may only appear as a secondary binding at a much higher concentration. It has been proposed that the aggregation of nisin–lipid II complexes at the membrane surface lead to the formation of a higher order macro-assembly that permeabilizes the bacterial membrane, where the apparent nisin : lipid II stoichiometry becomes 2 : 1.^46^

In comparison, lipid II binding of Txb apparently does not trigger pore formation. Nevertheless, this observation does not rule out the possibility that Txb–lipid II complexes cluster into a higher-order assembly at membrane surfaces without forming a pore, especially when the function of the mutation sensitive N-terminal residues remains unannotated. Interestingly, quantitative NMR analysis suggested that the cooperativity of substrate binding in Txb mainly depends on its N-terminal residues, which also showed somewhat lower substrate affinity than the main lipid II-binding residues at the C-terminus.^22^

### The pyrophosphate cage of teixobactin

In addition to Txb, several other antibiotics also target lipid II specifically.^47^–^49^ A number of those molecules have their structures resolved in complex with lipid II analogs, revealing relevant details of their substrate interactions. For example, nisin binds predominantly to the pyrophosphate,^37^ vancomycin binds the pentapeptide,^50^ and bacitracin binds the pyrophosphate with the requirement of two cationic cofactors.^51^

The lipid II binding interactions of Txb found in our simulations are distinct from those of vancomycin and bacitracin since Txb does not interact with the pentapeptide. In contrast to bacitracin, Txb phosphate binding is achieved through direct hydrogen bonds rather than coordinating pyrophosphate-bound cations.

To compare further with bacitracin, we analyzed the probability of ion coordination by Txb in simulation Systems 1 and 6 that exemplify the two binding poses. No significant ion residence at the lipid II-bound Txb occurred within a 2.5 Å cutoff of H–Cl^–^ or O–Na^+^ distances. Most transient ion contacts occur around the solution-exposed backbone carbonyl atoms and are only present for <5% of the total simulation time. In contrast to Txb, bacitracin utilizes two acidic side chains to coordinate the ion cofactors. Since Txb bears no acidic residue, it is unlikely that its substrate recognition is cofactor dependent.

Unexpectedly, the Txb–lipid II interactions we observed resemble the “pyrophosphate cage” described in the structures of nisin–lipid II complexes.^37^ Both antibiotics achieve specific lipid II binding by forming multiple hydrogen bonds coordinating the pyrophosphate moiety, which is mainly contributed by the amide groups of the peptide backbone within a cyclic structure. The pyrophosphate binding of nisin is provided primarily by its ring A and partially by the immediately connected ring B, where the two rings constitute a conserved structural motif shared by many lantibiotics.^37^ Lipid II binding of Txb also relies heavily on the backbone amides at the cyclodepsipeptide ring; however, Txb's ring structure bears little similarity to that of the lantibiotics other than the ability to surround an anionic entity. Interestingly, our hydrogen bond analysis of Systems 1 and 6 shows that the ester group of the cyclodepsipeptide ring provides no interaction with lipid II or other phospholipids, and is only partially hydrated at the ester carbonyl oxygen. Since this ester group appears responsible only for the ring cyclization, it is possible that the ester group can be replaced by an amide, or a thioether (as in a lantibiotic), or even a disulfide group to create functional Txb analogs.

The structure and chemical composition of Txb have drawn comparison with a completely unrelated compound,^48^,^52^ the peptide-based antibiotic hypeptin.^53^ Surprisingly, even though the two peptide sequences bear no similarity and little is known about the function and mechanism of hypeptin, the two compounds share several common structural features that might indicate their functional and mechanistic resemblance. Firstly, both have a cyclodepsipeptide ring of exactly the same size, and both rings are cyclized with an ester bond between the C-terminal carboxylate group and the side chain of a d-amino acid. Regardless of the residue composition of the cyclodepsipeptide ring, the backbone of this ring is the main component of the “pyrophosphate cage” of Txb. Secondly, both compounds carry a +2*e* net charge in solution, which is contributed by the N-terminal amino group and a side chain guanidinium group. The guanidinium group from the *allo*-End10 of Txb serves as a secondary phosphate-coordinator, which is possibly the role of the arginine side chain in hypeptin if it also binds lipid II. Finally, both antibiotics have a cluster of hydrophobic residues outside the cyclodepsipeptide ring, and the hydrophobic residues in Txb determine its membrane insertion. Based on the shared features, it was hypothesized that hypeptin also targets lipid II.^48^,^52^ If true, investigating the common lipid II interactions shared by Txb and hypeptin may lead to the development of a new class of pyrophosphate caging antibiotics.

### Implications on the inhibitory mechanism

The domain composition and arrangement of penicillin-binding proteins suggest that the peptidoglycan transpeptidation normally takes place in a soluble domain away from the membrane, whereas the transglycosylation step takes place in a periphery membrane domain near the membrane surface.^54^ The proximity of the Txb–lipid II complex to the membrane surface, as well as the exclusion of the lipid II pentapeptide from significant Txb interactions shown in our simulations, both suggest that Txb is unlikely to inhibit the transpeptidation. In addition, our simulation results also showed that Txb has little to no interaction with the terminal GlcNAc of lipid II (Fig. 4), nor does the binding of Txb affect the relative position of that GlcNAc with respect to the membrane surface (Fig. 3c). These observations suggest that, even in a Txb-bound lipid II, the terminal GlcNAc remains solution-exposed with unrestricted movement, and thus might be available for interactions with the glycosyltransferase and participate in the following step of the peptidoglycan growth (Fig. 5, dotted arrow). This one-step transglycosylation might be granted by the wide opening of the glycosyl acceptor site of the glycosyltransferase,^55^ with most interactions to the acceptor lipid II surrounding the disaccharide.^56^ Even if the Txb-bound lipid II can accept the growing polysaccharide chain, however, the reaction product would be trapped at the acceptor site and unable to enter the donor side due to its limited volume that cannot accommodate the bound Txb, thereby preventing further reaction with lipid II and arresting the cell wall synthesis (Fig. 5, dashed arrows).

## Conclusions

Through stepwise modeling and MD simulations, we captured two distinct binding poses of the Txb–lipid II complex that appear stable at a membrane surface. In both conformations, lipid II binding is achieved primarily by phosphate coordination near the C-terminal cyclodepsipeptide ring, while two hydrophobic residues in the middle of the peptide chain serve as the membrane anchor. The binding of lipid II-pyrophosphate takes place at the phosphate level of the membrane. No significant interaction is observed between Txb and the lipid II disaccharide/pentapeptide. Based on the Txb–lipid II binding poses, both the specific binding to lipid II phosphate and the non-specific binding to the membrane surface appear essential for Txb activity. The interactions and location of the Txb–lipid II complex suggest that the antibiotic is more likely to inhibit the transglycosylation step than the transpeptidation step of peptidoglycan synthesis.

## Conflicts of interest

There are no conflicts to declare.

## Supplementary Material

Supplementary information
