# Supplementary material for: Probing key elements of teixobactin–lipid II interactions in membranes
Source: Chem Sci. 2018 Jul 20;9(34):6997–7008. doi: 10.1039/c8sc02616e (PMC6124899; doi:10.1039/c8sc02616e)
Supplement: Supplementary file 1 [file SC-009-C8SC02616E-s001.pdf]

Electronic Supplementary Material (ESI) for Chemical Science.
This journal is © The Royal Society of Chemistry 2018

Probing Key Elements of Teixobactin-Lipid II Interactions in Membrane

Electronic Supplementary Information

Po-Chao Wen[1], Juan M. Vanegas[2], Susan B. Rempe[3], and Emad Tajkhorshid[1]

[1]Department of Biochemistry, Beckman Institute for Advanced Science and Technology, University of Illinois at Urbana-Champaign, Urbana, Illinois 61801, U.S.A.
[2]Department of Physics, University of Vermont, Burlington, VT 05405, U.S.A.
[3]Department of Nanobiology, Center for Biological and Engineering Sciences, Sandia National Laboratories, Albuquerque, NM 87185, U.S.A.

July 20, 2018

Computational Methods

The outline of procedures to model and simulate Txb-lipid II complex is described in the Result section and illustrated in Fig. 2 of the main article. Listed below are the detailed protocols to conduct system construction, MD simulations, and data analysis.

General Protocols of MD simulations

NAMD [1] (version 2.12) was used to perform MD simulations and the CHARMM36 force field was used to describe biological molecules, with parameter sets for proteins [2], lipids [3], carbohydrates [4], and lipid-linked oligosaccharides [5], which also include parameters from the CHARMM General Force Field, CGenFF [6]. The TIP3P [7] model was used for water since standard CHARMM force field parameters are developed based upon interactions with TIP3P water. Additional force field parameters were constructed using procedures described above for functional groups not included in existing CHARMM36 parameter sets.

All systems were simulated as NPT ensembles at constant temperature $T = 310\,\mathrm{K}$ and pressure $P = 1.01325\,\mathrm{bar}$ (N ~93,200 atoms for membrane systems containing Txb-lipid II complexes; $N = 73{,}398$ atoms for lipid II in membrane; and $N = 20{,}268$ atoms for Txb and lipid II head group in solution). The thermostat utilizes a Langevin dynamics with a damping coefficient $\gamma = 0.5\,\mathrm{ps}^{-1}$, and the Langevin piston Nosé-Hoover method [8, 9] was used for the barostat. In order to account for the different compressibility along the membrane surface from that of the aqueous solution, a semi-isotropic pressure coupling scheme was applied when a lipid bilayer was included in the simulation system, in which the pressure was only coupled at the xy dimensions. Simulations were integrated at 2-fs time steps except for the initial model preparation of the lipid bilayers, where a 1-fs time step was used. Pairwise non-bonded interactions were calculated using a 12 Å cutoff distance with a switching function employed between 10–12 Å; long-range electrostatic interactions were calculated using the particle mesh Ewald (PME) method [10]. Simulation trajectories were collected every 5 ps.

Development of Force Field Parameters

Several molecular components of Txb are not readily available for simulation in CHARMM36: the methylated N-terminus, the ester linkage between D-Thr8 and the C-terminus, and the side chain of L-*allo*-enduracididine (*allo*-End). Similarly, topology and parameters are required for lipid II at the N-acetylmuramate (MurNAc), its peptide link to the N-terminus of the pentapeptide, and the γ-peptide linkage within the pentapeptide chain. The paragraphs below describe the procedures to determine the topology and parameters of these chemical components.

Lipid II. The residue topology required for lipid II was developed solely based on molecular analogy. The saccharide MurNAc was created by merging the topology of N-acetylglucosamine (GlcNAc) with that of 2-methoxy-propionate, both of which are available from the CHARMM36 carbohydrate force field [4] (residue `AGLCNA` in `top_all36_carb.rtf` and residue `AMOP` in `toppar_all36_carb_model.str`, respectively). Parameters for the two non-proteinogenic peptide linkages in lipid II were adopted from the corresponding terms in the CHARMM36 protein force field [2], and partial charges were redistributed among the relevant atoms following the consistent trend among all peptide groups: N (-0.47 e), H (0.31 e), C (0.51 e), O (-0.51 e). Note that the novel

atom type CT2A was introduced into the CHARMM36 protein force field to address the χ_1/χ_2 dihedral rotations of anionic side chains. Since the formation of the γ-D-Glu peptide bond renders the side chain neutral, the carbon atoms of the D-Glu residue were re-typed as ordinary CT2 and assigned -0.18 e partial charges.

Teixobactin: N-terminus. The partial charges at the methylated N-terminus of Txb were assigned based on the analogy to the side chain of methylated lysine in the CHARMM36 protein force field [2] (residue MLYS in toppar_all36_prot_model.str), while the angle and dihedral terms were determined using the Force Field Toolkit (ffTK) [11]. The force constant of the CH_3–NH_2–C_α angle was fitted to the quantum mechanical Hessian calculation of N-isopropylmethylammonium ion, whereas the force constants of dihedrals associated with the NH_2–C_α bond were fitted to the QM dihedral scanning results of N-methylated D-Ala dipeptide at its ϕ dihedral. The program Gaussian 09 [12] was used to perform QM calculations of both compounds, where the 6-31G* basis set and the MP2 level of theory were used.

Teixobactin: Ester. Ester atoms at the D-Thr8–C-terminal linkage were assigned atom types from the esterization C-terminal patch of the CHARMM36 protein force field [2] (patch CT1 in top_all36_prot.rtf). The partial charges at this ester group were adopted from the ester groups in the CHARMM36 lipid force field [3] instead of the aforementioned protein patch since Txb is expected to partition into the phospholipid bilayers to interact with lipid II, and the dipole of this ester group should resemble more a phospholipid than a freely soluble ester (*e.g.*, methyl acetate, from which parameters of the CT1 patch were based upon).

Teixobactin: L-*allo*-enduracididine. The detailed procedures to develop parameters of *allo*-End side chain will be described in greater detail in another manuscript. In short, the standard topology/parameters for protein backbone atoms are preserved in *allo*-End, while the side chain parameters were optimized using ffTK [11]: firstly, partial charges and in-ring bond/angle/dihedral parameters were optimized according to QM calculations of a side chain analog (S)-4-methylimidazolidine-2-iminium, followed by optimization of $\chi1/\chi2$ dihedral parameters based on the potential energy surface obtained from a two dimensional QM dihedral scan, where the backbone ϕ/ψ dihedrals were fixed at angles corresponding to the cyclodepsipeptide ring geometry.

Construction and Simulation of Membrane Systems

To construct the simulation system of the membrane-bound lipid II, the coordinates of the lipid II head group were adopted from the solution structure of the nisin-lipid II complex (PDB: 1WCO [13], model #1 of 20) and the undecaprenyl tail appended. To generate the coordinates for the undecaprenyl group, atomic coordinates of the first 5 carbon atoms of the farnesyl pyrophosphate were preserved from the NMR structure, and the atomic coordinates of the rest of the undecaprenyl group were calculated according to the internal coordinates provided for undecaprenyl pyrophosphate [5] (with two dihedral internal coordinates corrected[1]). The generated full-length lipid II with

[1]The lipid-linked oligosaccharides stream file from the CHARMM36 force field contains two internal coordinates causing misplaced atoms: one creates a *trans*-isoprene at the 8th subunit and the other creates a methyl group where the three hydrogen atoms are separated by only 60° dihedral.

fully extended undecaprenyl tail was placed over a non-equilibrated bilayer of 1-palmitoyl-2-oleoyl-phosphatidylethanoamine (POPE), which was generated from the Membrane plugin of VMD (145 POPE molecules per leaflet, $\sim 100\times 100\,\text{Å}^2$ before equilibration, see Fig. 2 of main article). Due to the length of the fully extended undecaprenyl group, the lipid II head group was placed away from the membrane surface and only the last four isoprene subunits (#8–#11) were inserted into the top leaflet (in parallel to other non-equilibrated acyl chains of POPE). This POPE bilayer with a partially inserted lipid II was then solvated with ~100 mM NaCl solution into a $\sim 100\times 100\times 80\,\text{Å}^3$ periodic box. Similarly, systems exploring the membrane-bound conformations of the Txb/lipid II complex were constructed by extending the polyprenyl chain from the modeled head group complexes obtained from solution simulations (described later), followed by the same procedures of membrane insertion and solvation with a ~100 mM saline solution.

The equilibration of all membrane containing systems start with a "tail-melting" step for 0.5 ns, during which all atom coordinates remain fixed except for the acyl chains of the lipids and the undecaprenyl group of lipid II. The "melted" bilayers were then simulated without any constraint, during which the initially solution-exposed parts of the undecaprenyl group partitioned into the membrane rapidly (on the order of ~10 ns). The system with isolated membrane-bound lipid II was simulated for 1 μs, whereas systems including both Txb and lipid II were simulated for either 50 ns or 500 ns.

Modeling the Complex of Teixobactin and Lipid II

The construction of a preliminary model of an analog complex between the Arg10 mutant of Txb and a nerylneryl-pyrophosphoryl-N-acetyl-α-D-glucosaminide ($C_{20}H_{33}-P_2O_7{}^{2-}-$GlcNAc) molecule will be described in greater detail in a separate manuscript. In brief, the two molecules were first simulated for 100 ns in aqueous solution with a harmonic restraint applied between the center of mass of each molecule. The trajectories were clustered to identify the top 5 representative conformations, and the complexes of each conformation were further simulated for 500 ns in equilibrium without the harmonic restraint. The interaction energy between the two molecules in all the 5 trajectories was analyzed to determine the most energetically favorable complex conformation, and the lowest energy conformation was selected as the basis to construct the initial model of the complex between the wild type Txb and the head group of lipid II.

The modeled analog complex was superimposed onto the 1 μs trajectory of membrane-bound lipid II from earlier simulations at the pyrophosphate group, and the conformation of the lipid II head group with the fewest steric clashes to the Arg10-Txb (no atomic contact within 1.8 Å distance between Arg10-Txb and either the dissacharide or the pentapeptide; atomic contacts to the undecaprenylpyrophosphate ignored) was selected for further modeling along with the "bound" Arg10-Txb. The undecaprenyl chain of lipid II in the modeled complex was truncated except for the first isoprene subunit, and the Arg10 residue of the mutant Txb was replaced with the wild-type *allo*-End residue using the coordinates from QM-optimized conformation of the *allo*-End dipeptide. The newly placed *allo*-End side chain was then minimized for 3,000 steps in vacuum while all other atoms of the modeled complex were fixed. The resulting binary complex between the wild-type Txb and the dimethylallyl derivative of lipid II was solvated into a $60\times 60\times 60\,\text{Å}^3$ periodic box containing ~100 mM NaCl solution.

To sample the interactions between Txb and the lipid II head group and identify the most probable bound forms, the modeled complex in solution was subjected to 1 μs of adaptive tempering simulations [14], where the system temperature fluctuated in the range of 298–450 K (in

304 bins). During the adaptive tempering simulations, the branching carbon of the dimethylallyl group remained fixed at its initial coordinates near the origin of the periodic box (*i.e.*, only the Txb molecule was freely diffusing, Fig. S3), to limit translation of the lipid II head group to allow convenient *post hoc* analysis.

The trajectories of the 1 μs adaptive tempering simulations were divided into many short sections, each accounting for one encounter between the two molecules. An encounter is defined when any atom (including hydrogens) of one molecule is located within 3.0 Å from any atom of the other molecule and lasts until no atom can be found within a 3.0 Å cutoff. A representative complex model was generated for each encounter, based on the snapshot with lowest potential energy between the two molecules during the encounter. The resulting complex models were then ranked based their potential energy, as well as the lifetime of the encounter they were representing (*i.e.*, the lowest interaction energy and the life time of the transient complex). A total of 10 highest ranked complex models were selected to have the undecaprenyl chain extended from the dimethylallyl group, inserted into and equilibrated with POPE bilayers following the procedures described above. All of these membrane systems were simulated for 50 ns, each in two replicas using the same initial structure but different initial velocities generated with different random seeds. Two of the 10 systems where most of the initial contacts were maintained after 50 ns were extended to 500 ns for both replicas from the same initial structure.

Trajectory Analysis

The MD trajectories were analyzed using VMD [15] and its plugins. The interaction energy between Txb and lipid II was calculated using the NAMD Energy plugin of VMD. The relative positions of chemical components with respect to the lipid bilayer were computed as follows: the trajectories were first re-centered to the center of mass of all the POPE molecules, and the membrane surface was determined using the mean z-coordinate of all the phosphorus atoms of POPE molecules in the *cis* leaflet, while the z-position of each component was represented by either the center of mass of an amino acid (Txb or lipid II pentapeptide), the geometric center of a pyranose ring (MurNAc and GlcNAc), or the phosphorus coordinate of a phosphate moiety. The lifetime of a hydrogen bond was recorded with the starting and termination time points, using a criterion of 2.5 Å distance cutoff between the proton and its acceptor, according to the analysis by R. E. Hubbard [16, 17]. Here in the simulation systems, the polar proton refers to any hydrogen atom with partial charge $>0.16\,e$, while oxygen atoms are the only hydrogen bond acceptors present. For simplicity of computation, the relative orientations between the hydrogen bond donors and acceptors (angles and dihedrals) were not taken into account in the criterion. The apparent $t_{1/2}$ of a hydrogen bond was determined by the median lifetime of all the hydrogen bonds formed between the same proton:acceptor pair. Hydrophobic contacts were calculated in similar manner using the 2.5 Å distance cutoff between non-polar protons from either Txb or lipid II.

References

[1] James C. Phillips, Rosemary Braun, Wei Wang, James Gumbart, Emad Tajkhorshid, Elizabeth Villa, Christophe Chipot, Robert D. Skeel, Laxmikant Kale, and Klaus Schulten. Scalable molecular dynamics with NAMD. *J. Comp. Chem.*, 26:1781–1802, 2005.

[2] Robert B. Best, Xiao Zhu, Jihyun Shim, Pedro E. M. Lopes, Jeetain Mittal, Michael Feig, and

Alexander D. MacKerell. Optimization of the additive CHARMM all-atom protein force field targeting improved sampling of the backbone ϕ, ψ and side-chain χ_1 and χ_2 dihedral angles. *J. Chem. Theory Comput.*, 8(9):3257–3273, 2012.

[3] J. B. Klauda, R. M. Venable, J. A. Freites, J. W. O'Connor, D. J. Tobias, C. Mondragon-Ramirez, I. Vorobyov, A. D. MacKerell Jr., and R. W. Pastor. Update of the CHARMM all-atom additive force field for lipids: Validation on six lipid types. *J. Phys. Chem. B*, 114(23):7830–7843, 2010.

[4] Olgun Guvench, Sairam S. Mallajosyula, E. Prabhu Raman, Elizabeth Hatcher, Kenno Vanommeslaeghe, Theresa J. Foster, Francis W. Jamison, and Alexander D. MacKerell. CHARMM additive all-atom force field for carbohydrate derivatives and its utility in polysaccharide and carbohydrate protein modeling. *J. Chem. Theory Comput.*, 7(10):3162–3180, 2011.

[5] Nathan R. Kern, Hui Sun Lee, Emilia L. Wu, Soohyung Park, Kenno Vanommeslaeghe, Jr. Alexander D. MacKerell, Jeffery B. Klauda, Sunhwan Jo, and Wonpil Im. Lipid-linked oligosaccharides in membranes sample conformations that facilitate binding to oligosaccharyltransferase. *Biophys. J.*, 107(8):1885–1895, 2014.

[6] K. Vanommeslaeghe, E. Hatcher, C. Acharya, S. Kundu, S. Zhong, J. Shim, E. Darian, O. Guvench, P. Lopes, I. Vorobyov, and A. D. MacKerell, Jr. CHARMM General Force Field: A force field for drug-like molecules compatible with the CHARMM all-atom additive biological force fields. *J. Comp. Chem.*, 31(4):671–690, 2010.

[7] William L. Jorgensen, Jayaraman Chandrasekhar, Jeffry D. Madura, Roger W. Impey, and Michael L. Klein. Comparison of simple potential functions for simulating liquid water. *J. Chem. Phys.*, 79(2):926–935, 1983.

[8] G. J. Martyna, D. J. Tobias, and M. L. Klein. Constant pressure molecular dynamics algorithms. *J. Chem. Phys.*, 101(5):4177–4189, 1994.

[9] Scott E. Feller, Yuhong Zhang, Richard W. Pastor, and Bernard R. Brooks. Constant pressure molecular dynamics simulation: The Langevin piston method. *J. Chem. Phys.*, 103(11):4613–4621, 1995.

[10] Tom Darden, Darrin York, and Lee G. Pedersen. Particle mesh Ewald: An $N{\cdot}\log(N)$ method for Ewald sums in large systems. *J. Chem. Phys.*, 98(12):10089–10092, 1993.

[11] Christopher G. Mayne, Jan Saam, Klaus Schulten, Emad Tajkhorshid, and James C. Gumbart. Rapid parameterization of small molecules using the Force Field Toolkit. *J. Comp. Chem.*, 34:2757–2770, 2013.

[12] M. J. Frisch, G. W. Trucks, H. B. Schlegel, G. E. Scuseria, M. A. Robb, J. R. Cheeseman, G. Scalmani, V. Barone, B. Mennucci, G. A. Petersson, H. Nakatsuji, M. Caricato, X. Li, H. P. Hratchian, A. F. Izmaylov, J. Bloino, G. Zheng, J. L. Sonnenberg, M. Hada, M. Ehara, K. Toyota, R. Fukuda, J. Hasegawa, M. Ishida, T. Nakajima, Y. Honda, O. Kitao, H. Nakai, T. Vreven, J. A. Montgomery, Jr., J. E. Peralta, F. Ogliaro, M. Bearpark, J. J. Heyd, E. Brothers, K. N. Kudin, V. N. Staroverov, R. Kobayashi, J. Normand, K. Raghavachari, A. Rendell, J. C. Burant, S. S. Iyengar, J. Tomasi, M. Cossi, N. Rega, J. M. Millam, M. Klene, J. E. Knox,

J. B. Cross, V. Bakken, C. Adamo, J. Jaramillo, R. Gomperts, R. E. Stratmann, O. Yazyev, A. J. Austin, R. Cammi, C. Pomelli, J. W. Ochterski, R. L. Martin, K. Morokuma, V. G. Zakrzewski, G. A. Voth, P. Salvador, J. J. Dannenberg, S. Dapprich, A. D. Daniels, Ö. Farkas, J. B. Foresman, J. V. Ortiz, J. Cioslowski, and D. J. Fox. *Gaussian 09 Revision B.01*, 2009. Gaussian Inc. Wallingford CT 2009.

[13] Shang-Te D. Hsu, Eefjan Breukink, Eugene Tischenko, Mandy A. G. Lutters, Ben de Kruijff, Robert Kaptein, Alexandre M. J. J. Bonvin, and Nico A J van Nuland. The nisin-lipid II complex reveals a pyrophosphate cage that provides a blueprint for novel antibiotics. *Nat. Struct. Mol. Biol.*, 11(10):963–967, 2004.

[14] Cheng Zhang and Jianpeng Ma. Enhanced sampling and applications in protein folding in explicit solvent. *J. Chem. Phys.*, 132:244101, 2010.

[15] William Humphrey, Andrew Dalke, and Klaus Schulten. VMD – Visual Molecular Dynamics. *J. Mol. Graphics*, 14(1):33–38, 1996.

[16] E. N. Baker and R. E. Hubbard. Hydrogen bonding in globular proteins. *Prog. Biophys. Mol. Biol.*, 44(2):97–179, 1984.

[17] Rodrick E. Hubbard and Muhammad Kamran Haider. Hydrogen bonds in proteins: role and strength. In *Encyclopedia of Life Sciences (eLS)*. John Wiley & Sons, Ltd, Chichester, UK, 2010.

Supporting Figures

Figure S1. List of hydrogen bonds between Txb and lipid II at $t = 0$ or 50 ns of the 10 modeled complexes simulated in membrane. The 10 modeled complexes were constructed based on the 10 highest ranked conformations captured in the 1 μs adaptive tempering simulation in solution. The hydrogen bonds are categorized by the involvement of either the cyclodepsipeptide ring amindes, the *allo*-End10 side chain, or others, and are listed in three blocks, respectively. For clarity, hydrogen bonds at $t = 50$ ns of the third block are listed only if it is retained from $t = 0$ (thus Y/N retention) or involves phosphate binding (P1 or P2). Hydrogen bonds are listed as residue names of the donor/acceptor, where P1 denotes any hydrogen-bond accepting oxygen atom of the polyprenyl-connecting phosphate (including the bridging oxygen between the two phosphates), and P2 denotes the other oxygen atoms of the saccharide-connecting phosphate (including the bridging oxygen between phosphate and MurNAc). Hydrogen bonds retained at $t = 50$ ns of both runs are highlighted in bold.

Figure S2. Atom numbering and naming schemes for parts of Txb and lipid II. Atom names are derived from standard nomenclature of amino acids and saccharide rings.

Figure S3. Close-up view of Txb-lipid II hydrogen bonds in the top 10 modeled complexes from the 1 μs adaptive tempering simulations in solution (the end of step iii and the beginning of step iv). The general atomic coloring scheme in the main article is followed. See Fig. S1 ($t = 0$) for the list of hydrogen bonds illustrated here.

Figure S4. Hydrophobic/non-polar contact probabilities between components of Txb and lipid II in both runs of Systems 1 and 6. Contact probabilities were calculated between $t = 50$ ns and $t = 500$ ns using a 2.5 Å cut-off distance between non-polar hydrogen atoms of Txb and lipid II. The color scheme of Fig.4a of the main article is followed. SC, side-chain.

Complex Rank	H-bonds by Ring Amides			H-Bonds by *allo*-End10 Side Chain			Additional Hydrogen Bonds			
	t = 0	t = 50 ns		t = 0	t = 50 ns		Initial H-bonds (t = 0)		Retention (t = 50 ns)	
		Run 1	Run 2		Run 1	Run 2	Teixobactin	Lipid II	Run 1	Run 2
**1**	**P2**	**P2**	**P1,P2**	**P1**<br>**γ-D-Glu2**	**P1**<br>**γ-D-Glu2**	**P1**<br>**γ-D-Glu2**	**D-Phe1**<br>D-Gln4<br>**Ser7**	**P1,MurNAc**<br>MurNAc<br>**P2**	**(P1 only)**<br>N<br>**Y**	**(P1 only)**<br>N<br>**Y**
2	γ-D-Glu2	γ-D-Glu2	—	P1,P2<br>Ala1	P2<br>MurNAc,Ala1	—<br>—	D-Phe1<br>Ser7	P1,MurNAc<br>γ-D-Glu2	N<br>N	N<br>N
3	P2	—	—	**P1**<br>γ-D-Glu2	**P1,P2**<br>—	**P2**<br>—	—	—	—	—
4	**P1**	**P1,P2**	**P1**	γ-D-Glu2	—	P1	D-Phe1<br>Ser7	P2<br>P1	N<br>(P2)	N<br>N
5	—	—	—	P1,P2	—	—	D-Phe1<br>Ile2	P1,P2<br>P2	N<br>N	Y<br>Y
**6**	**P1**	**P1**	**P1**	**P1,P2**	**P2**	**P2**	D-Phe1<br>Ser3<br>D-Gln4	D-Ala4,D-Ala5<br>γ-D-Glu2<br>P2	N<br>N<br>N	N<br>N<br>N
7	P2	P2	—	**P1**	**P1**	**P1**	D-Phe1<br>D-Gln4<br>Ser7	D-Ala4,D-Ala5<br>γ-D-Glu2,Lys3<br>P2	N<br>N<br>Y	N<br>N<br>N
8	**P1**	**P1**	**P1**	**P2**	**P1,P2**	**P2**	D-Phe1<br>Ser3<br>D-Gln4<br>**Ser7**	D-Ala5<br>GlcNAc<br>γ-D-Glu2<br>**P1**	N<br>N<br>N<br>**Y**	N<br>N<br>N<br>**Y**
9	**P1**	**P1**	**P1**	**P2**<br>MurNAc<br>γ-D-Glu2	**P1**<br>—<br>—	**P1**<br>—<br>—	Ser3<br>**Ser7**	MurNAc<br>**P1**	N<br>**Y**	N<br>**(P2)**
10	**P1**	**P2**	**P1**	P1	P1	—	D-Phe1,Ile2	D-Ala5	N	N

Figure S1: Teixobactin-lipid II hydrogen bonds.

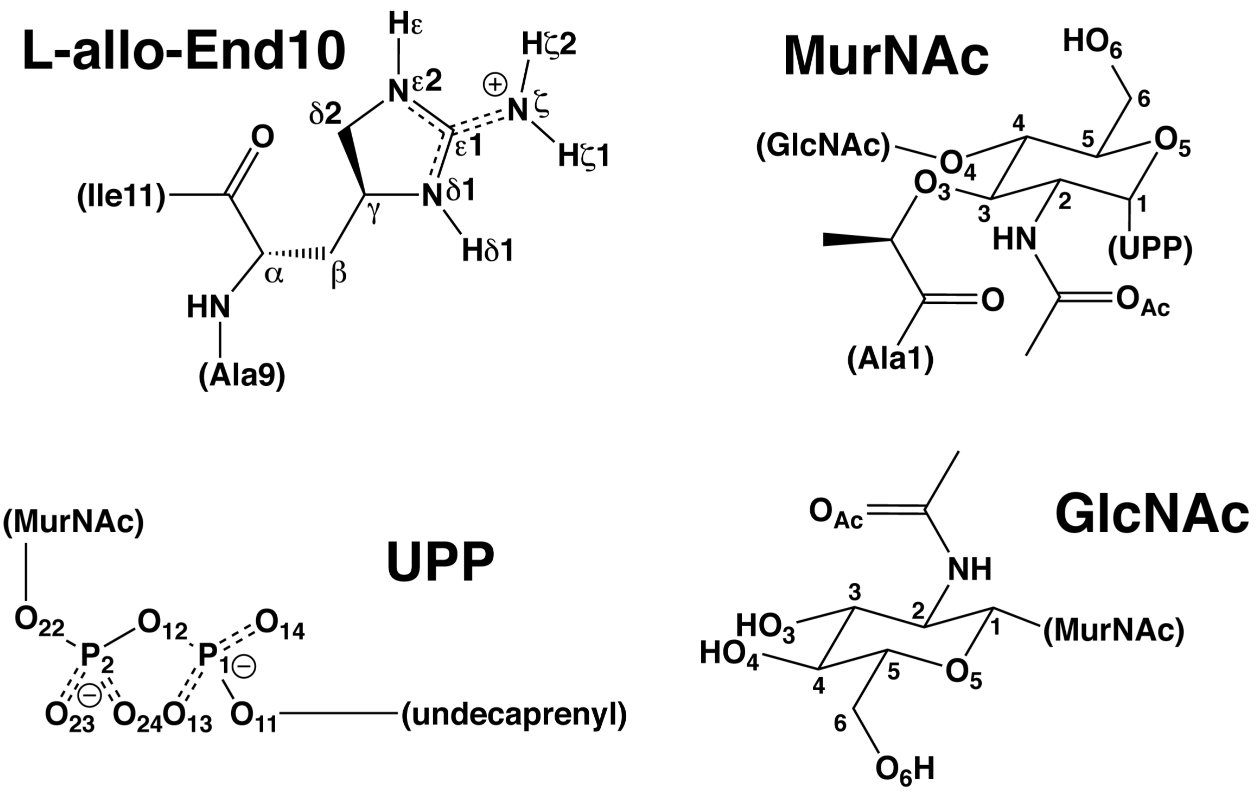

Figure S2: Atom names of some components of teixobactin and lipid II.

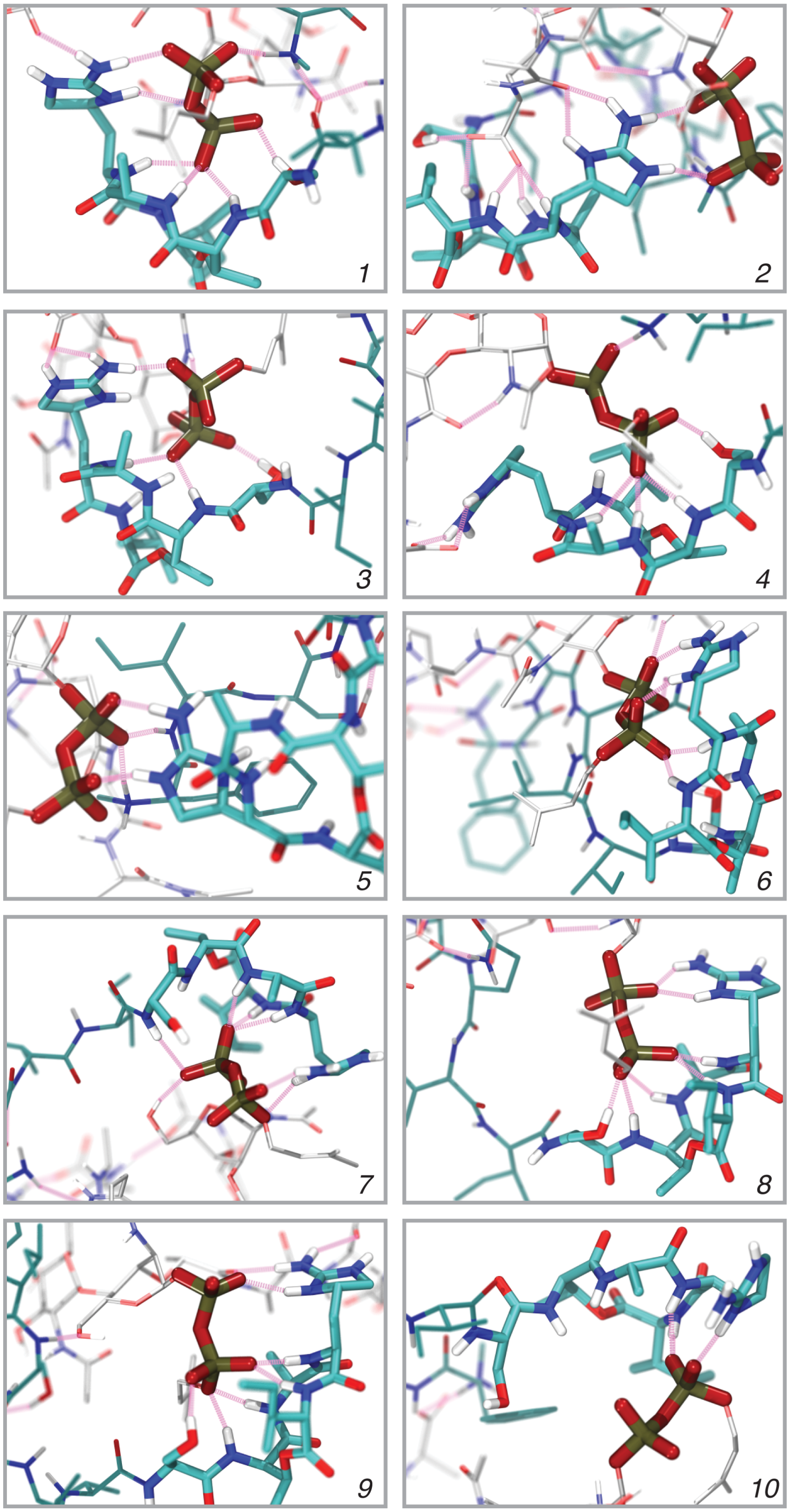

Figure S3: Teixobactin-lipid II hydrogen bonds in the top 10 complexes of solution simulations.

**System 1-1**	**Hydrophobic Contact to Lipid II (%)**			**System 6-1**	**Hydrophobic Contact to Lipid II (%)**		
**Teixobactin**	**Isoprenes**	**Disaccharide**	**Pentapeptide**	**Teixobactin**	**Isoprenes**	**Disaccharide**	**Pentapeptide**
D-Phe1 (H_α)	0.000	0.143	0.002	D-Phe1 (H_α)	0.000	0.000	0.019
D-Phe1 (sc)	1.744	11.868	18.234	D-Phe1 (sc)	1.618	0.216	2.030
Ile2 (H_α)	0.000	5.818	1.592	Ile2 (H_α)	0.000	0.000	0.000
Ile2 (sc)	0.260	35.498	13.103	Ile2 (sc)	4.229	0.001	0.010
Ser3 (H_α)	0.000	28.520	0.000	Ser3 (H_α)	0.000	0.000	0.026
Ser3 (sc)	0.000	5.172	0.174	Ser3 (sc)	0.000	0.000	0.410
D-Gln4 (H_α)	0.039	0.001	0.000	D-Gln4 (H_α)	0.000	1.080	0.001
D-Gln4 (sc)	0.780	55.244	0.000	D-Gln4 (sc)	1.136	55.058	38.652
D-*allo*-Ile5 (H_α)	0.000	0.041	0.000	D-*allo*-Ile5 (H_α)	0.000	0.002	0.000
D-*allo*-Ile5 (sc)	15.041	1.362	0.000	D-*allo*-Ile5 (sc)	26.617	8.949	0.009
Ile6 (H_α)	0.422	0.000	0.000	Ile6 (H_α)	3.661	30.177	0.000
Ile6 (sc)	88.447	0.024	0.000	Ile6 (sc)	78.364	0.826	0.038
Ser7 (H_α)	50.958	0.003	0.000	Ser7 (H_α)	0.000	0.000	0.158
Ser7 (sc)	0.014	44.427	0.000	Ser7 (sc)	0.000	59.102	2.002
D-Thr8 (H_α)	26.419	0.000	0.000	D-Thr8 (H_α)	0.016	0.000	0.420
D-Thr8 (sc)	6.283	0.211	0.000	D-Thr8 (sc)	0.682	0.000	0.838
Ala9 (H_α)	0.753	0.000	0.000	Ala9 (H_α)	0.000	0.042	0.537
Ala9 (sc)	74.000	0.000	0.000	Ala9 (sc)	0.000	4.776	1.986
allo-End10 (H_α)	0.000	0.000	0.002	*allo*-End10 (H_α)	0.000	0.000	0.000
allo-End10 (sc)	0.029	6.579	26.644	*allo*-End10 (sc)	28.282	0.029	0.000
Ile11 (H_α)	0.001	0.000	0.004	Ile11 (H_α)	0.027	0.000	0.000
Ile11 (sc)	0.000	76.734	14.643	Ile11 (sc)	88.621	0.000	0.000
**System 1-2**	**Hydrophobic Contact to Lipid II (%)**			**System 6-2**	**Hydrophobic Contact to Lipid II (%)**		
**Teixobactin**	**Isoprenes**	**Disaccharide**	**Pentapeptide**	**Teixobactin**	**Isoprenes**	**Disaccharide**	**Pentapeptide**
D-Phe1 (H_α)	0.000	0.009	0.013	D-Phe1 (H_α)	0.000	0.000	0.017
D-Phe1 (sc)	17.912	50.502	26.938	D-Phe1 (sc)	6.230	2.619	9.740
Ile2 (H_α)	0.000	2.103	0.009	Ile2 (H_α)	0.123	0.649	0.176
Ile2 (sc)	0.000	49.718	14.363	Ile2 (sc)	14.560	6.541	5.547
Ser3 (H_α)	0.000	2.173	0.008	Ser3 (H_α)	0.000	0.000	0.332
Ser3 (sc)	0.000	3.497	0.047	Ser3 (sc)	0.183	0.030	8.674
D-Gln4 (H_α)	0.000	0.000	0.000	D-Gln4 (H_α)	0.431	0.018	1.210
D-Gln4 (sc)	0.000	0.260	0.000	D-Gln4 (sc)	0.006	14.796	15.781
D-*allo*-Ile5 (H_α)	0.002	0.000	0.000	D-*allo*-Ile5 (H_α)	0.000	0.000	0.000
D-*allo*-Ile5 (sc)	6.559	0.000	0.000	D-*allo*-Ile5 (sc)	39.591	24.651	15.703
Ile6 (H_α)	0.001	0.000	0.000	Ile6 (H_α)	2.603	1.339	0.011
Ile6 (sc)	91.160	0.000	0.000	Ile6 (sc)	82.591	3.351	0.000
Ser7 (H_α)	52.421	0.000	0.000	Ser7 (H_α)	0.121	0.000	0.029
Ser7 (sc)	0.098	0.000	0.000	Ser7 (sc)	0.000	0.647	12.452
D-Thr8 (H_α)	20.441	0.000	0.000	D-Thr8 (H_α)	0.002	0.000	0.000
D-Thr8 (sc)	6.350	0.000	0.000	D-Thr8 (sc)	2.444	0.000	0.000
Ala9 (H_α)	0.859	0.000	0.000	Ala9 (H_α)	0.000	0.000	0.000
Ala9 (sc)	68.118	0.000	0.000	Ala9 (sc)	0.000	0.000	0.094
allo-End10 (H_α)	0.000	0.000	0.001	*allo*-End10 (H_α)	0.000	0.000	0.000
allo-End10 (sc)	0.000	60.098	12.879	*allo*-End10 (sc)	22.404	5.561	0.000
Ile11 (H_α)	0.000	0.000	0.000	Ile11 (H_α)	0.003	0.000	0.000
Ile11 (sc)	0.011	76.733	0.051	Ile11 (sc)	81.729	0.000	0.000

Figure S4: Probability of non-polar/hydrophobic contacts between Txb and lipid II components.
